# Clinical and Genetic Characterization of a Family with Tooth Agenesis Carrying a Novel *LEF1* Variant

**DOI:** 10.3390/dj14100659

**Published:** 2026-10-10

**Authors:** Ludovica Fortuna, Roberto Bertozzi, Valentina Trevisan, Chiara Leoni, Angelo Minucci, Maurizio Genuardi, Gaetano Isola, Cristina Grippaudo, Elisabetta Tabolacci

**Affiliations:** 1 Departmental Unit of Molecular and Genomic Diagnostics, Fondazione Policlinico Universitario A. Gemelli IRCCS, 00168 Rome, Italy; ludovica.fortuna@guest.policlinicogemelli.it (L.F.); angelo.minucci@policlinicogemelli.it (A.M.); 2 Genomics Core Facility, Gemelli Science and Technology Park (G-STeP), Fondazione Policlinico Universitario A. Gemelli IRCCS, 00168 Rome, Italy; 3 Center for Rare Diseases, Department of Woman and Child Health and Public Health, Fondazione Policlinico Universitario A. Gemelli IRCCS, 00168 Rome, Italy; valentina.trevisan@policlinicogemelli.it (V.T.); chiara.leoni@policlinicogemelli.it (C.L.); 4 Department of Life Sciences and Public Health, Section of Genomic Medicine, Catholic University of the Sacred Heart, 00168 Rome, Italy; maurizio.genuardi@unicatt.it (M.G.); elisabetta.tabolacci@unicatt.it (E.T.); 5 Complex Operational Unit of Medical Genetics, Fondazione Policlinico Universitario A. Gemelli IRCCS, 00168 Rome, Italy; 6 Unit of Periodontology, Department of General Surgery and Surgical-Medical Specialties, School of Dentistry, University of Catania, 95123 Catania, Italy; gaetano.isola@unict.it; 7 International Research Center on Periodontal and Systemic Health “PerioHealth”, University of Catania, 95123 Catania, Italy; 8 UOC Clinica Odontoiatrica, Dipartimento di Neuroscienze, Organi di Senso e Torace, Fondazione Policlinico Universitario A. Gemelli IRCCS, 00168 Rome, Italy; 9 Dipartimento Universitario Testa Collo ed Organi di Senso, Università Cattolica del Sacro Cuore, 00168 Rome, Italy

**Keywords:** tooth agenesis, *LEF1* gene, whole exome sequencing

## Abstract

**Background**: Tooth agenesis is one of the most common developmental anomalies of the human dentition and may occur either as an isolated trait or as part of a syndromic condition. Advances in genomic technologies have facilitated the identification of genes involved in odontogenesis, including *LEF1* (Lymphoid Enhancer-Binding Factor 1), a key transcription factor in the WNT signaling pathway. **Methods**: Whole exome sequencing (WES) was performed in a proband with non-syndromic tooth agenesis to investigate the underlying genetic cause. Parental segregation analysis was performed through Sanger sequencing, and *in silico* prediction tools were used to assess the potential clinical relevance of the identified variant. A review of the current literature on *LEF1*-associated dental phenotypes was also conducted. **Results**: WES identified a novel heterozygous *LEF1* variant, c.903G>C, p.(Lys301Asn). The variant co-segregated with the phenotype, being detected in the affected father and absent in the unaffected mother. Although the available evidence is currently insufficient to establish pathogenicity, computational predictions and the known biological role of LEF1 in odontogenesis support its potential functional significance. **Conclusions**: This study reports a novel *LEF1* variant identified in a family with familial non-syndromic tooth agenesis and highlights the importance of integrating genomic findings with clinical and segregation data to inform variant interpretation in hereditary dental anomalies. Further functional and independent genetic evidence will be required to establish the clinical significance of this variant.

## 1. Introduction

Tooth agenesis is one of the most common developmental anomalies in humans, affecting approximately 2–10% of the population when third molars are excluded [1]. Dental agenesis is classified based on the number and position of missing teeth, and depending on the severity of the clinical situation, it can cause alterations in dental occlusion and facial growth [2]. The condition may occur as an isolated trait (non-syndromic hypodontia or oligodontia) or as part of a broader syndromic disorder involving ectodermal derivatives [3]. Genetic studies have identified several genes involved in tooth development, including *WNT10A*, *AXIN2*, *MSX1*, *PAX9*, and *LEF1*, highlighting the critical role of epithelial–mesenchymal signaling during odontogenesis [4,5].

*LEF1* (Lymphoid Enhancer-Binding Factor 1) encodes a transcription factor of the TCF/LEF family that acts as a key mediator of canonical WNT/β-catenin signaling. During tooth development, *LEF1* is expressed in both the dental epithelium and mesenchyme and is essential for the reciprocal epithelial–mesenchymal interactions that drive tooth morphogenesis [6]. Experimental studies in animal models have demonstrated that disruption of LEF1 function results in arrested tooth development, underscoring its crucial role in odontogenesis [7].

Recently, both monoallelic and biallelic pathogenic variants in *LEF1* have been associated with human disease, expanding the phenotypic and inheritance spectrum of *LEF1*-related disorders [8]. Heterozygous pathogenic variants have been associated with an ectodermal dysplasia phenotype characterized predominantly by tooth agenesis and taurodontism, together with variable abnormalities of other ectodermal derivatives, including hair and sweat glands [8]. In contrast, biallelic *LEF1* variants have been reported in individuals with a more severe multisystemic phenotype, including ectodermal dysplasia and limb malformations, consistent with a broader disruption of WNT signaling [8]. These findings indicate that *LEF1*-associated disease can result from both monoallelic and biallelic variants, with the clinical presentation potentially influenced by the allelic state and the nature of the underlying variant.

These observations are consistent with experimental data demonstrating that *LEF1* is a key transcriptional effector required for epithelial–mesenchymal interactions during organogenesis, particularly in odontogenesis, where it is expressed in both dental epithelium and mesenchyme and is essential for progression beyond early tooth developmental stages [6,7]. Moreover, functional disruption of WNT pathway components, including *LEF1*, has been extensively linked to dental developmental defects in both animal models and human genetic disorders, further supporting its role in human tooth formation [9]. However, as *LEF1* has only recently emerged as a disease-associated gene in humans, the variant landscape of *LEF1*-related disorders remains limited and still evolving, with relatively few pathogenic variants reported to date. Consequently, the full phenotypic spectrum and genotype–phenotype correlations remain incompletely defined and continue to be refined as additional cases are reported in the literature. Moreover, available evidence is largely derived from a limited number of families and case series, limiting the establishment of robust genotype–phenotype correlations. Therefore, the pathogenicity of newly identified variants, particularly rare missense substitutions, frequently remains uncertain due to the absence of functional studies, limited segregation data, and lack of recurrence in unrelated affected individuals. Continued reporting of novel cases and systematic functional characterization will therefore be essential to improve variant classification and to better define the phenotypic spectrum associated with *LEF1* dysfunction.

In this study, we report a family with congenital dental anomalies carrying a heterozygous *LEF1* missense variant, c.903G>C, p.(Lys301Asn), identified through Whole Exome Sequencing (WES). The variant was subsequently shown to segregate with the phenotype within the family, being present in the affected father and absent in the unaffected mother, compatible with, but not proving, a causal role. To our knowledge, this specific missense change has not been previously reported in population databases or in scientific literature. By expanding the genotypic spectrum of *LEF1* variants and further delineating the associated clinical phenotype, our findings contribute to the growing understanding of *LEF1*-related dental developmental disorders and provide additional evidence to refine potential genotype-phenotype correlations.

## 2. Materials and Methods

The proband was enrolled at the Medical Genetics Unit of the Fondazione Policlinico Universitario A. Gemelli IRCCS, Rome, within the framework of the Gen&Rare research project, aimed at the genomic profiling of genetic and rare diseases to personalize diagnostic, therapeutic, and preventive pathways. The proband and his parents underwent clinical evaluations and genetic counseling. Observation was based on orthopanoramic radiographs (OPT) and on intra- and extra-oral photographs. The study protocol was written in accordance with the Declaration of Helsinki, and the study was approved by the local Ethic Committee of the Catholic University of Sacred Heart of Rome (Protocol No.0000161/26, ID 6987). Samples were collected after signed informed consent of the proband and their parents. Subsequently, biological samples were collected, including two peripheral blood samples in EDTA-containing tubes and one saliva sample. One peripheral blood sample and the saliva sample were stored in the Biobank of the Fondazione Policlinico Universitario A. Gemelli IRCCS for potential future investigations. The second peripheral blood sample was used directly for molecular analysis.

Genomic DNA was extracted from the latter peripheral blood sample using the automated EZ2 Connect Automated DNA/RNA Purification System (QIAGEN, Milan, Italy), according to the manufacturer’s instructions. Following quality control assessment, including DNA quantification and fragmentation analysis, genomic DNA underwent NGS analysis. Specifically, whole exome sequencing (WES) was performed using the Illumina DNA Prep with Exome 2.5 Enrichment kit for library preparation, target enrichment, and capture, with automated processing carried out on Hamilton instrumentation. The resulting libraries were subsequently sequenced on a NovaSeq 6000 System (Illumina, San Diego, CA, USA). Sequencing data generated in FASTQ format were processed using the DRAGEN Bio-IT Platform v4.3 (Illumina, San Diego, CA, USA). The analysis generates BAM files, containing reads aligned to the reference genome (GRCh38), and Variant Call Format (VCF) files, in which all detected variants are annotated. VCF files were subsequently analyzed using the Illumina Emedgene platform, an artificial intelligence-based tool for variant prioritization. Variant prioritization considered population allele frequency (MAF < 1%), sequencing quality (read depth and genotype quality ≥20×), predicted functional impact, phenotype relevance. Copy-number variant (CNV) analysis was performed from the WES data using the DRAGEN CNV Calling pipeline (Illumina, San Diego, CA, USA). As WES was performed, genes known to be associated with tooth agenesis, including *WNT10A*, *MSX1*, *PAX9*, and *AXIN2*, were included in the analysis, and no pathogenic variants were identified in these genes. Variant ranking is achieved through the automated integration of patient clinical information, scientific literature, public databases (including ClinVar, gnomAD, dbSNP, dbVAR, DGV, and DECIPHER), and variant-specific characteristics. Clinical features were encoded using Human Phenotype Ontology (HPO) terms. Specifically, “Tooth agenesis” was encoded using the HPO term HP:0009804. The Emedgene platform enables the identification and prioritization of variants that are most consistent with the patient’s phenotype, disease presentation, candidate genes, and expected mode of inheritance. In the final stage of the analysis, identified variants were interpreted and classified according to the ACMG/AMP guidelines [10], integrating molecular evidence with the patient’s clinical phenotype, gene–disease validity, and expected inheritance pattern.

Specifically, the variant reported in the present study exhibited optimal technical parameters, including sequencing depth (coverage ≥30×), high variant-calling quality, and variant allele frequency (VAF) consistent with the expected zygosity state, thereby fulfilling the laboratory’s internal reliability criteria. To further support the clinical and molecular interpretation of the variant identified through WES, segregation analysis was performed by Sanger sequencing (Applied Biosystems 3500, Monza, Italy) in the proband and his parents.

Structural analysis of the LEF1 protein was performed using the experimental NMR structure of the LEF1 HMG domain deposited in the Protein Data Bank (PDB ID: 2LEF) as the structural template. The p.Lys301Asn substitution was introduced into the corresponding structure, and the resulting model was subjected to energy minimization to remove local steric clashes and optimize the geometry around the substituted residue. The wild-type and mutant structures were subsequently inspected and visualized to assess the local structural context of the variant. Residues shown in the structural representation were included for spatial context only, without inferring specific molecular interactions or functional effects.

## 3. Results

A 9-year-and-3-month-old male patient was brought by his parents for an orthodontic examination in the outpatient clinic. The primary reason for the visit was the unsightly appearance of large gaps between the upper and lower incisors. Medical history was unremarkable, with no reported systemic comorbidities or past illnesses.

**Figure 1:**
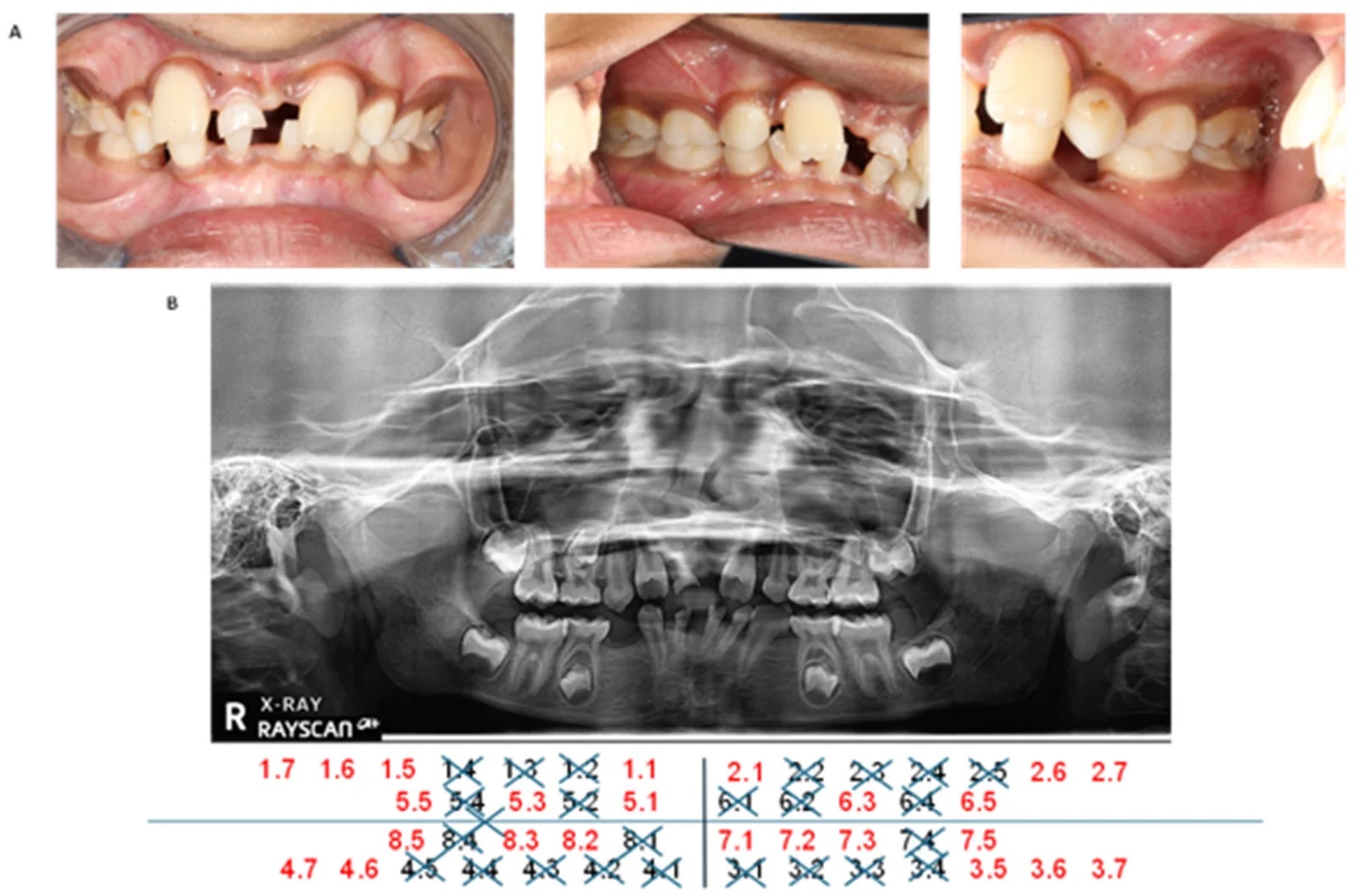
Photos and OPT of the proband. (**A**) Patient’s appearance at the first orthodontic visit. (**B**) Orthopantomography of the patient’s dental arches at the start of treatment. In the dental formula, the red teeth represent the present teeth in the arch; the black and crossed-out teeth represent the missing teeth of the deciduous and permanent series.

The dental occlusion showed the absence of the lateral incisors, and the first deciduous molars in the upper arch, with the permanence of a deciduous central incisor. One deciduous incisor was missing in the lower arch, while the other three were present in the arch. The first two deciduous molars were also absent in the lower arch. The mandibular permanent incisors were missing. The first permanent molars had erupted in both arches. Measurement of the pulp chamber of the first permanent molars, according to the Shifman and Chanannel index, classifies these teeth as affected by hypotaurodontism, since the value obtained from the index is equal to 20 [11]. The clinical significance is that mild taurodontism is present. The patient’s face was symmetrical, with a low lower third and a convex profile. The tongue was protruded between the incisors, with an anterior lingual thrust during swallowing. The lower deciduous incisors were not mobile but were strongly inclined toward the lips (Figure 1A). An orthopantomography (OPT) and lateral teleradiographs of the skull were requested. The orthopantomography showed the absence of the following permanent elements: 1.2–1.3–1.4–2.2–2.3–2.4–2.5–3.1–3.2–3.3–3.4–4.1–4.2–4.3–4.4 (Figure 1B).

Cephalometric values measured on lateral teleradiograph of the skull were within the normal range, except for the angle between the mandibular plane and the Frankfurt plane (FMA) which measured 13°, indicating hypodivergence of the mandible. Despite the agenesis of eight permanent teeth in the mandibular arch, no immediate orthodontic treatment was considered necessary at this stage because almost all deciduous teeth were still present and clinically stable, maintaining the mandibular arch without significant spacing. In contrast, treatment of the maxillary arch was considered appropriate because the presence of the persistent deciduous central incisor and the maxillary diastema affected both function and aesthetics, including phonetics and swallowing. A comprehensive treatment plan for both arches will be reconsidered as the remaining deciduous teeth exfoliate.

**Figure 2:**
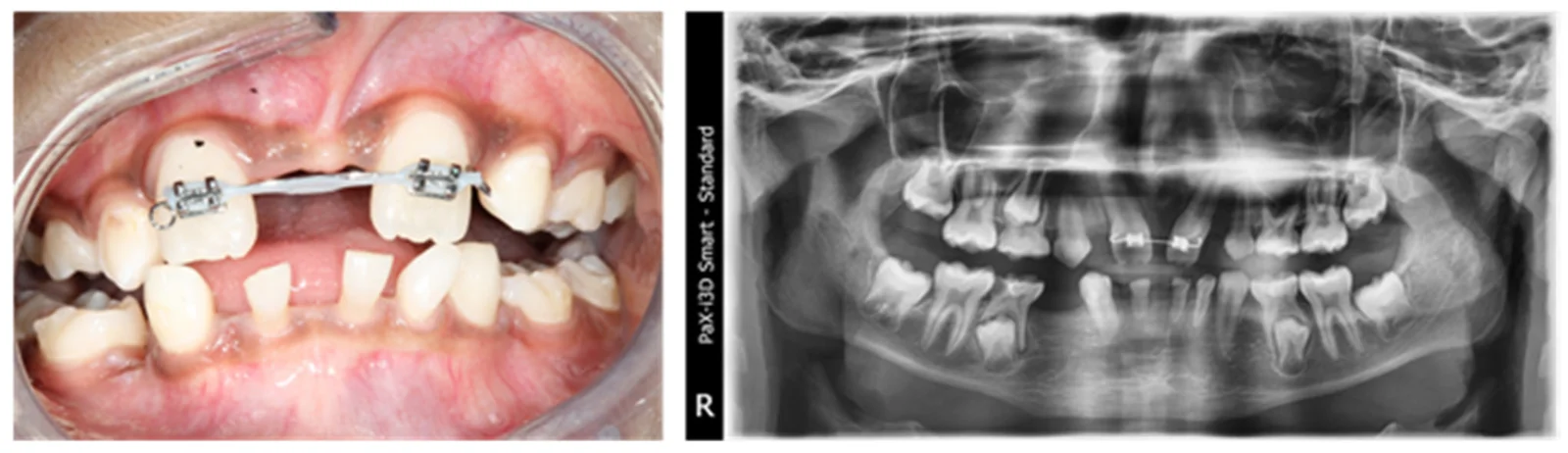
Orthodontic treatment after extraction of the deciduous central incisor.

The orthodontic treatment plan included the extraction of the upper deciduous central incisor and the bringing together of the permanent incisors to reduce the diastema and create space where the lateral incisors should have been. At the end of this phase, the patient will be followed up and will be fitted with a removable upper retainer with two resin elements replacing the lateral incisors. A prosthetic rehabilitation plan for the occlusion will then be developed (Figure 2).

Concurrent with orthodontic treatment, the patient underwent speech therapy sessions to improve tongue posture and function.

**Figure 3:**
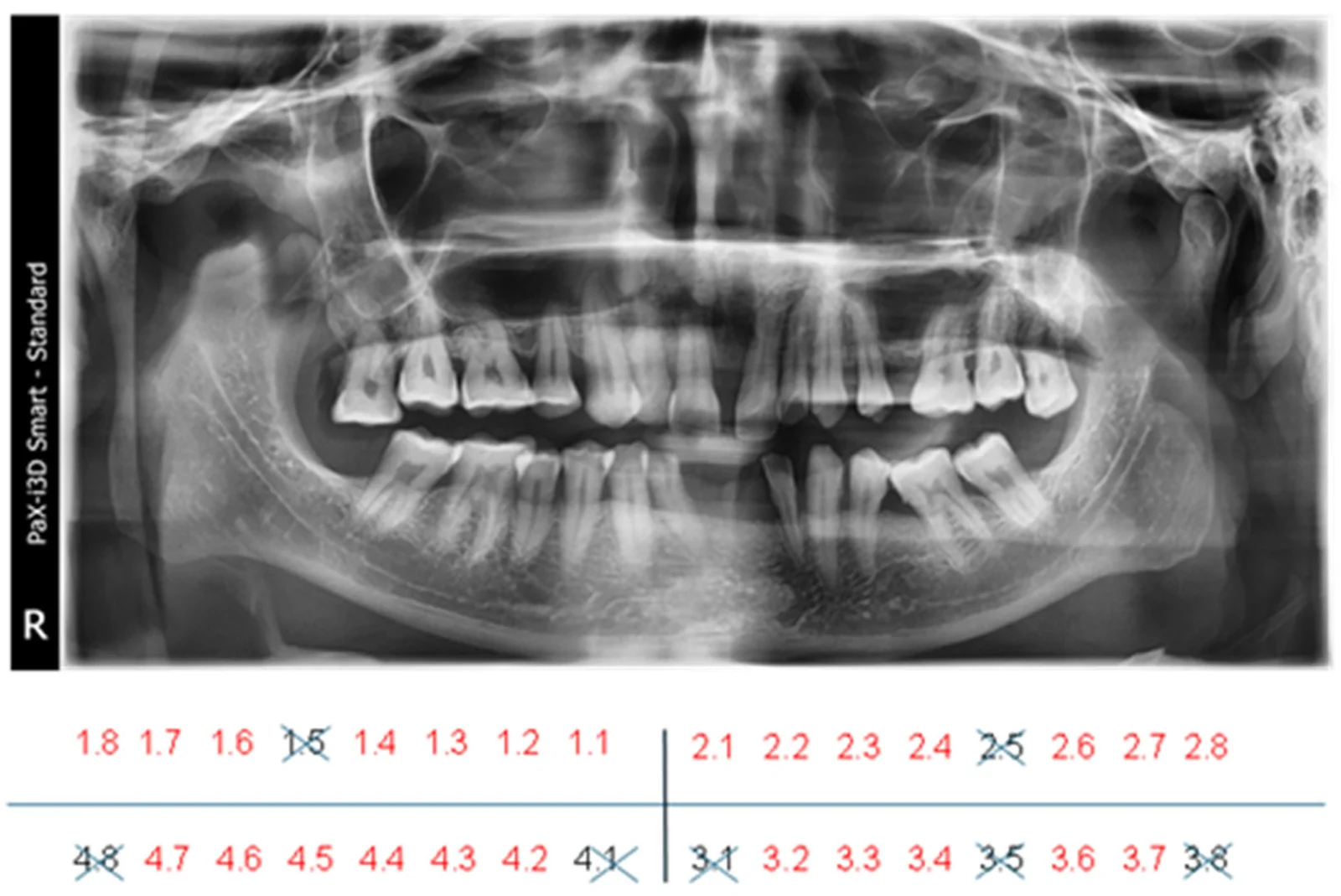
Orthopantomography of the father. The black and crossed-out teeth in the dental formula are missing due to agenesis.

Family history was notable for dental agenesis in the father (Figure 3), whereas the mother did not report similar dental abnormalities. The following teeth are missing: 1.5, 2.5, 3.5, 3.1, 3.8, 4.1, 4.8. The proband’s father reports that he has never had any tooth extractions and he wears a removable denture to replace his agenesis central incisors. An examination of his orthopantomography shows that the lower molars exhibit the root and pulp anatomy most indicative of taurodontism. Applying the Shifman–Chanannel index, the measurements indicate values equal to 30 for teeth 4.7, 4.6 and 3.7, border line between hypotaurodontism and mesotaurodontism. The element 3.6 shows a value of 20 hypotaurodontism). However, measurements on orthopantomography have limitations due to calibration errors, because a three-dimensional structure can only be visualized in 2D, so we present a descriptive analysis.

- Tooth 47 (lower right second molar): It presents a prominent, vertically elongated pulp chamber and short, fused roots.
- Tooth 46 (lower right first molar): It also shows a depressed pulp floor and reduced root separation.
- Tooth 36 (lower left first molar): It shows a vertical extension of the pulp chamber that requires a formal measurement.
- Element 37 (left lower second molar) presents a strongly compressed anatomy and an elongated pulp chamber, similar to its counterpart in the opposite quadrant.

No additional relevant ectodermal abnormalities were documented at the time of evaluation in the proband and his father. In particular, clinical assessment of the proband and his affected father did not reveal apparent abnormalities of the skin, hair, or nails, and no history of abnormal sweating was reported.

Genomic testing through WES was performed in the proband (the affected child) and did not identify pathogenic or likely pathogenic variants in known disease-associated genes (including *WNT10A*, *MSX1*, *PAX9* and *AXIN2*), nor clinically significant copy number variations involving genes or genomic regions associated with the phenotype.

**Figure 4:**
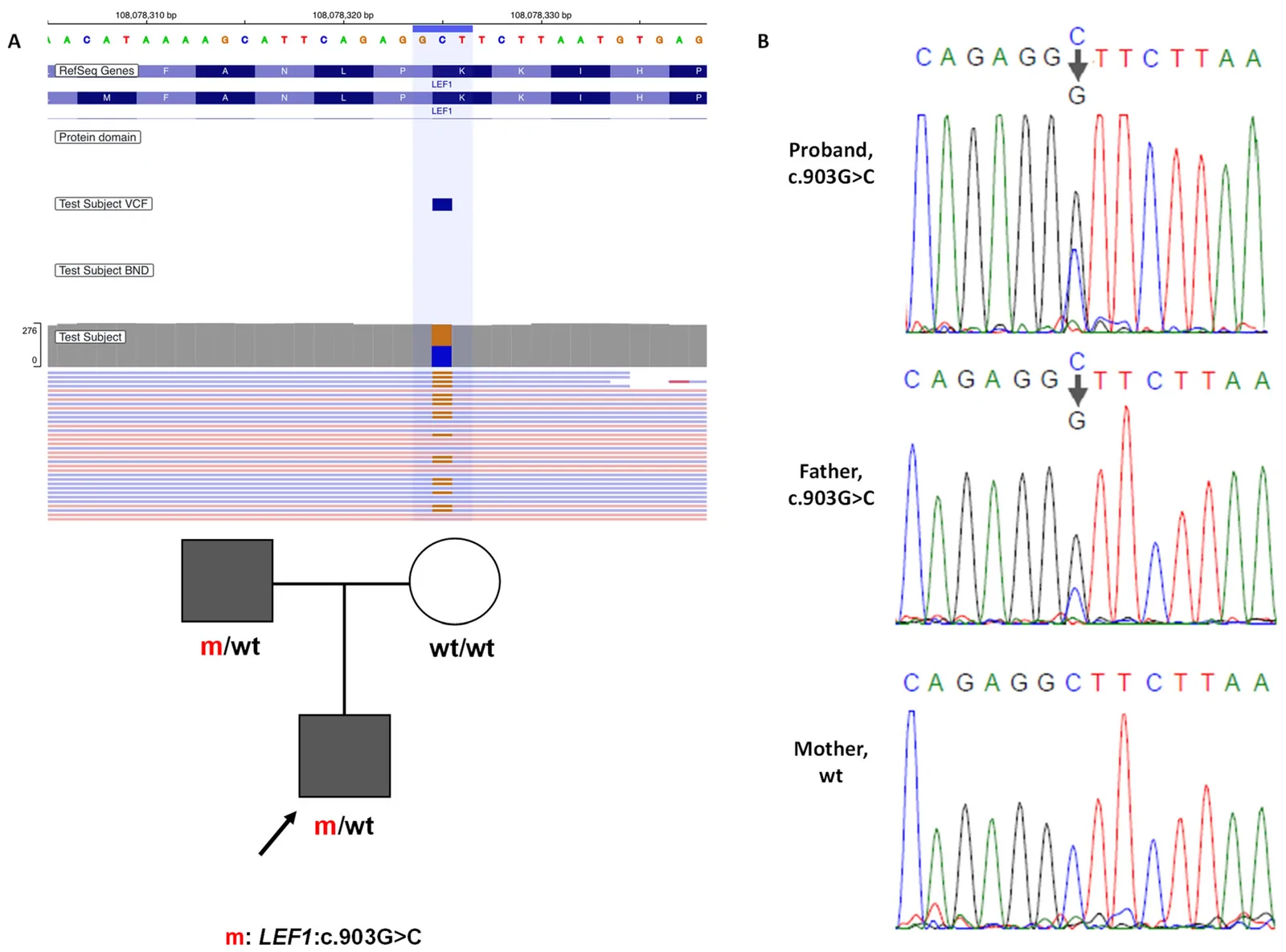
Molecular analysis of *LEF1* variant. (**A**) IGV analysis of the proband variant found in WES. The variant is G>C in the template strand. The pedigree is shown below. Filled symbols indicate affected individuals carrying the variant. The arrow indicates the proband. (**B**) Segregation analysis through Sanger sequencing in the proband and his parents. Electropherogram of tract of exon 8 in which the G>C transversion was found in the proband and his father (**upper** and **middle** panels), while it is absent (wild type, wt) in the unaffected mother (**bottom** panel).

However, a heterozygous missense variant in *LEF1* (NM_016269.5) was identified, c.903G>C, resulting in the amino acid substitution p.(Lys301Asn) (Figure 4).

The variant was not reported on the Genome Aggregation Database (gnomAD v4.1.1; last access 3 August 2026), suggesting that it is extremely rare in the general population. Furthermore, it was not present in ClinVar (last access 3 August 2026), indicating that no previous clinical submissions or interpretations have been described.

Segregation analysis demonstrated that the variant was absent in his unaffected mother and present in heterozygous state in his father, who also exhibited tooth agenesis (see Figure 4). The younger sibling is currently too young to undergo a reliable phenotypic assessment, as permanent dentition has not yet fully erupted. Both clinical evaluation and molecular testing are planned in the future to further clarify segregation within the family. This pattern is compatible with a potential autosomal dominant inheritance mechanism, although the limited number of informative relatives precludes definitive segregation analysis.

**Figure 5:**
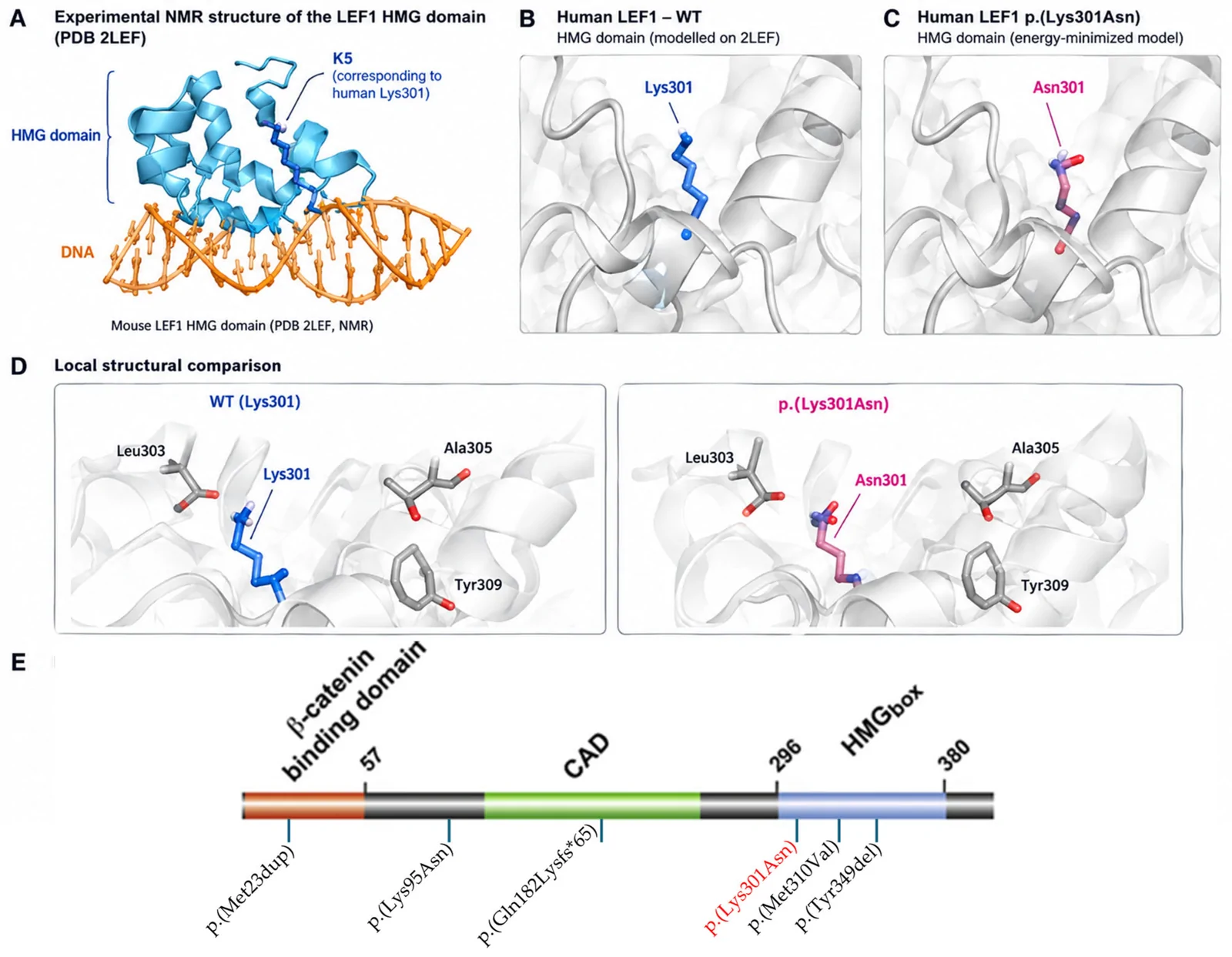
Structural analysis of the LEF1 p.(Lys301Asn) variant. (**A**) Experimental NMR structure of the mouse LEF1 HMG domain in complex with DNA (PDB 2LEF). The residue corresponding to human Lys301 is highlighted. (**B**) Structural representation of the human LEF1 HMG domain showing the position of Lys301. (**C**) Model of the human LEF1 p.(Lys301Asn) variant generated using the experimental HMG-domain structure as a structural reference and subjected to energy minimization. (**D**) Close-up comparison of the local structural environment of Lys301 in the wild-type protein and Asn301 in the energy-minimized mutant model. Neighboring residues are shown for structural context. (**E**) Schematic representation of the LEF1 protein domains, including the β-catenin-binding domain, the context-dependent activation domain (CAD), and the HMG-box DNA-binding domain. The position of the p.(Lys301Asn) variant is indicated within the HMG-box domain (in red). The figure also includes the other pathogenic or likely pathogenic variants associated with a dental phenotype, as reported in Table 1.

According to the ClinGen semi-quantitative Bayesian framework for ACMG/AMP variant interpretation, the *LEF1* c.903G>C, p.(Lys301Asn) variant yields a cumulative score of +3.0. The variant is absent from gnomAD, supporting PM2 at the Supporting level (+1 point), while the REVEL score of 0.81 supports PP3 at the Moderate level (+2 points). The available segregation data support PP1 at the Supporting level (+1 point). Although the variant co-segregates with the phenotype in the available family, the single informative meiosis is insufficient to support the application of PP1_Supporting criteria. PM1 was considered because the variant is located within the LEF1 HMG domain but was not applied because currently available evidence is insufficient to establish this region as a well-defined mutational hotspot or critical functional domain devoid of benign variation (Figure 5). Thus, the resulting cumulative Bayesian score is +3 points, corresponding to a Variant of Uncertain Significance (VUS, Class 3). Further functional and segregation studies will be required to clarify the clinical significance of this variant.

**Table 1:** Main *LEF1* variants associated with human developmental disorders associated and not associated with tooth agenesis.

Variant Description	Variant Type	Protein Region/Domain	Classification	Inheritance	Dental Phenotype	Associated Findings	Mechanism/Functional Evidence	Reference
4q25 microdeletion	Whole gene deletion	absent	n.a.	*de novo*	Severe oligodontia (22/28 permanent teeth missing in one patient; 19/28 in another)	Ectodermal dysplasia with skeletal abnormalities	Haploinsufficiency	Lévy et al., 2020 [12]
c.544del, p.(Gln182Lysfs*65)	Frameshift	Central regulatory region, truncating variant	Pathogenic	Segregates with phenotype	Agenesis of 2 teeth	Ectodermal dysplasia	Predicted loss-of-function	Dufour et al., 2022 [8]
c.69_71dupGAT, p.(Met23dup)	In-frame duplication	N-terminal, β-catenin-binding domain	Likely pathogenic	Homozygous (parents heterozygous and unaffected)	Oligodontia	Severe ectodermal dysplasia with ectrodactyly	~95 altered WNT pathway transcriptional profile	Dufour et al., 2022 [8]
c.1045_1047del, p.(Tyr349del)	In-frame deletion	HMG DNA-binding domain	Pathogenic	*de novo*	Tooth agenesis (2 teeth)	Ectodermal dysplasia with limb malformation	Altered subcellular localization, protein instability and aggregation	Dufour et al., 2022 [8]
c.928A>G, p.(Met310Val)	Missense	HMG DNA-binding domain	Likely pathogenic	Heterozygous; segregating with phenotype in two families	Variable (mainly periodontal/abnormal tooth implantation rather than severe agenesis)	Ectodermal dysplasia with predominantly radial ray defects	Proposed impaired DNA binding	Dufour et al., 2022 [8]
c.285G>C, p.(Lys95Asn)	Missense	N-terminal/centre region	Likely pathogenic	n.r.	Hypodontia/oligodontia	n.r.	Identified in a patient with non syndromic tooth agenesis, support a role in human odontogenesis	Biedziak et al., 2022 [4]

n.a., not available; n.r., not reported.

## 4. Discussion

Among the genes implicated in the molecular pathogenesis of tooth agenesis, *LEF1* has recently emerged as a key regulator of WNT/β-catenin-mediated epithelial–mesenchymal interactions during odontogenesis. A downstream effector of canonical WNT signaling, *LEF1* encodes a DNA-binding transcription factor essential for the transcriptional programs that orchestrate tooth development [13,14,15].

In this context, the identification of a novel missense variant in *LEF1* is of particular interest. The p.(Lys301Asn) substitution affects amino acid residue 301, within the highly conserved High Mobility Group (HMG) domain DNA-binding region of the protein, a region critical for sequence-specific DNA recognition and transcriptional regulation during organogenesis, including tooth development. The affected lysine residue is evolutionarily conserved across vertebrate species, and its substitution with asparagine introduces a change in charge and physiochemical proprieties that could impair DNA binding affinity and downstream transcriptional activity (Figure 5).

*In silico* analysis further support a potentially deleterious effect of the variant with PolyPhen-2 (https://genetics.bwh.harvard.edu/pph2/, accessed on 3 August 2026), SIFT (http://www.sift-dna.org/, accessed on 3 August 2026), REVEL (https://sites.google.com/site/revelgenomics/, accessed on 3 August 2026) (score 0.81, moderate) and AlphaMissense (https://alphamissense.hegelab.org/, accessed on 3 August 2026) (score 1, strong) predicting functional impairment, although computational evidence alone is insufficient for definitive classification. Several lines of evidence support the biological plausibility of pathogenicity; however, functional studies are required to confirm the functional impact of the variant and provide definitive evidence of its pathogenic effect. The variant is ultrarare from population databases, affects a highly conserved residue within a critical functional domain and segregates in affected family members. In addition, *LEF1* has already been established as a disease gene associated with autosomal dominant dental developmental disorders [16].

Experimental and clinical evidence further reinforce its relevance in odontogenesis. *LEF1* is expressed in both epithelial and mesenchymal compartments of developing teeth and mediates epithelial–mesenchymal signaling essential for normal tooth morphogenesis. Accordingly, loss-of-function models in mice exhibit severe defects in tooth formation, highlighting the essential role of *LEF1* during early developmental stages [17]. In humans, heterozygous variants in *LEF1* have been associated with tooth agenesis and taurodontism, expanding the phenotypic spectrum of LEF-related disorders confirming its role in dental development [12,18,19].

To our knowledge, the c.903G>C, p.(Lys301Asn) variant has not been previously reported in the scientific literature, and it is ultrarare in population databases. While its clinical significance cannot be definitively established, the combined genetic, evolutionary, structural and segregation evidence supports its consideration as candidate variant requiring functional validation. Further functional studies and the identification of additional unrelated cases will be critical to clarify its pathogenic role.

The limited number of reported *LEF1* variants suggests a broad phenotypic spectrum, ranging from non-syndromic tooth agenesis to ectodermal dysplasia with severe limb abnormalities (Table 1).

The available data indicates that the phenotype may depend on both the type of variant and its position within functional regions of the protein. Loss-of-function variants and deletions involving *LEF1* have mainly been associated with ectodermal and limb abnormalities, supporting a dosage-sensitive role of LEF1 during development. Conversely, missense variants affecting specific functional regions may result in more variable phenotypes. The novel p.(Lys301Asn) variant identified in our patient affects the HMG DNA-binding domain (Figure 5) of LEF1, implicated in DNA binding and transcriptional activity. Notably, the nearby p.(Met310Val) variant has been associated with *LEF1*-related developmental abnormalities, supporting the functional relevance of this region. The substitution of lysine with asparagine may alter local molecular interactions and potentially affect LEF1 function, as suggested by our structural analysis (Figure 5A–D). The predominant dental phenotype observed in our patient, in the absence of major limb or generalized ectodermal abnormalities, may suggest a variant-specific and potentially partial impairment of LEF1 activity. Although the currently available evidence does not allow definitive genotype–phenotype correlations, our findings expand the phenotypic and molecular spectrum associated with *LEF1* and further support its role in human tooth development.

## 5. Limitations and Future Perspectives

This study has several limitations that should be acknowledged. First, the analysis was performed in a single family, and no independent cases or additional families with *LEF1*-related tooth agenesis were available for comparison. Second, no functional data is currently available to support a causal role of the identified *LEF1* variant. In addition, the phenotypic assessment of the mother was incomplete, and the younger sibling could not be evaluated. These limitations preclude definitive conclusions regarding the pathogenicity of the variant. Functional studies, including *in vitro* assessment of the p.(Lys301Asn) variant, evaluation of LEF1 protein expression and stability, and analysis of its transcriptional activity and effects on Wnt/β-catenin signaling, will be required to clarify its potential contribution to the phenotype.

## 6. Conclusions

We describe a novel heterozygous missense *LEF1* variant, c.903G>C, p.(Lys301Asn), identified in a family with tooth agenesis. Although currently classified as a variant of uncertain significance, multiple converging lines of evidence—including its absence from population databases, localization within the HMG DNA-binding domain, evolutionary conservation, predicted functional effect, and familial segregation—support its potential clinical relevance. Additional functional and clinical evidence will be required to confirm its pathogenicity and further refine genotype–phenotype correlations in *LEF*-related dental disorders. This report further expands the molecular spectrum of tooth agenesis and contributes to the growing understanding of its genetic basis.

## Data Availability

All data generated or analyzed in this study are included within the article.

## References

[1] Meade M.J., Dreyer C.W. (2023). Tooth agenesis: An overview of diagnosis, aetiology and management. Jpn. Dent. Sci. Rev..

[2] Letra A., Chiquet B., Hansen-Kiss E., Menezes S., Hunter E., Adam M.P., Bick S., Mirzaa G.M., Pagon R.A., Wallace S.E., Amemiya A. (2021). Nonsyndromic Tooth Agenesis Overview. GeneReviews.

[3] Vastardis H. (2000). The genetics of human tooth agenesis: New discoveries for understanding dental anomalies. Am. J. Orthod. Dentofac. Orthop..

[4] Biedziak B., Firlej E., Dąbrowska J., Bogdanowicz A., Zadurska M., Mostowska A. (2022). Novel Candidate Genes for Non-Syndromic Tooth Agenesis Identified Using Targeted Next-Generation Sequencing. J. Clin. Med..

[5] Modafferi C., Tucci I., Bogliardi F.M., Gimondo E., Chiurazzi P., Tabolacci E., Grippaudo C. (2025). Genetic Aspects of Tooth Agenesis. Genes.

[6] Cadigan K.M., Nusse R. (1997). Wnt signaling: A common theme in animal development. Genes Dev..

[7] Sarkar L., Sharpe P.T. (1999). Expression of Wnt signalling pathway genes during tooth development. Mech. Dev..

[8] Dufour W., Alawbathani S., Jourdain A.S., Asif M., Baujat G., Becker C., Budde B., Gallacher L., Georgomanolis T., Ghoumid J. (2022). Monoallelic and biallelic variants in *LEF1* are associated with a new syndrome combining ectodermal dysplasia and limb malformations caused by altered WNT signaling. Genet Med..

[9] Balic A., Thesleff I. (2015). Tissue Interactions Regulating Tooth Development and Renewal. Curr. Top. Dev. Biol..

[10] Richards S., Aziz N., Bale S., Bick D., Das S., Gastier-Foster J., Grody W.W., Hegde M., Lyon E., Spector E. (2015). Standards and guidelines for the interpretation of sequence variants: A joint consensus recommendation of the American College of Medical Genetics and Genomics and the Association for Molecular Pathology. Genet Med..

[11] Shifman A., Chanannel I. (1978). Prevalence of taurodontism found in radiographic dental examination of 1,200 young adult Israeli patients. Community Dent. Oral Epidemiol..

[12] Lévy J., Capri Y., Rachid M., Dupont C., Vermeesch J.R., Devriendt K., Verloes A., Tabet A.C., Bailleul-Forestier I. (2020). LEF1 haploinsufficiency causes ectodermal dysplasia. Clin. Genet..

[13] Lee D.J., Won H.J., Shin J.O. (2026). Integrated Genetic Networks and Epigenetic Regulation in Tooth Development and Maturation. Cells.

[14] Eliason S., Sharp T., Sweat M., Sweat Y.Y., Amendt B.A. (2020). Ectodermal Organ Development Is Regulated by a microRNA-26b-Lef-1-Wnt Signaling Axis. Front Physiol..

[15] Sasaki T., Ito Y., Xu X., Han J., Bringas P., Maeda T., Slavkin H.C., Grosschedl R., Chai Y. (2005). LEF1 is a critical epithelial survival factor during tooth morphogenesis. Dev. Biol..

[16] Zhang H., Gong X., Xu X., Wang X., Sun Y. (2023). Tooth number abnormality: From bench to bedside. Int. J. Oral Sci..

[17] van Genderen C., Okamura R.M., Fariñas I., Quo R.G., Parslow T.G., Bruhn L., Grosschedl R. (1994). Development of several organs that require inductive epithelial-mesenchymal interactions is impaired in LEF-1-deficient mice. Genes Dev..

[18] de Lau W., Clevers H. (2001). LEF1 turns over a new leaf. Nat. Genet..

[19] Chetty M., Roomaney I.A., Beighton P. (2021). Taurodontism in dental genetics. BDJ Open.

